# Predictive value of serum neurofilament light chain for cognitive impairment in Parkinson’s disease

**DOI:** 10.3389/fnagi.2024.1465016

**Published:** 2024-12-05

**Authors:** Lihua Gu, Pengcheng Zhang, Rui Gao, Hao Shu, Pan Wang

**Affiliations:** ^1^Department of Neurology, Tianjin Huanhu Hospital, Tianjin, China; ^2^Academy of Military Medical Sciences, Academy of Military Sciences, Tianjin, China; ^3^Department of Neurology, the Fourth Affiliated Hospital of Nanjing Medical University, Nanjing, Jiangsu, China

**Keywords:** cognitive impairment, neurofilament light chain, Parkinson’s disease, serum, cohort

## Abstract

**Background:**

Neurofilament light chain (NfL) has recently emerged as a key indicator of neurodegeneration. In this study, our hypothesis is that the levels of blood-derived NfL and its accumulation during the Parkinson’s disease (PD) progression could serve as a potential biomarker for predicting subsequent cognitive decline. To investigate this, we conducted a study utilizing a large single-center cohort.

**Methods:**

The study included 193 participants, consisting of 106 cognitively normal PD (PD-CN) patients and 87 normal controls (NC) individuals. Serum NfL concentrations were measured. PD patients were followed up for clinical assessment at an average of 2 ± 0.6 years.

**Results:**

The serum NfL levels were significantly higher in PD-CN patients compared to NC. PD-CN patients and NC at follow-up time exhibited higher serum NfL levels compared to those at baseline. PD patients with high serum NfL levels were found to have a higher likelihood of transitioning from normal cognition to mild cognitive impairment (MCI) or dementia (Hazard ratio (HR) 1.107, 95% confidence intervals (CI) 1.010–1.213, *p* = 0.030). The area under the curve (AUC) for PD-CN conversion to MCI or dementia at follow-up time was determined to be 0.684 (95% CI 0.569–0.799).

**Conclusion:**

In conclusion, our study found that PD patients have significantly higher levels of serum NfL compared to individuals without PD. Furthermore, serum NfL levels increase as PD progresses and can predict cognitive impairment within a 2-year timeframe. Serum NfL may serve as a feasible, non-invasive biomarker of cognitive progression in PD. However, further studies and functional experiments are needed to validate these findings.

## 1 Introduction

Parkinson’s disease (PD) is a prevalent neurodegenerative disease worldwide, affecting 8 to 18 individuals per 100,000 each year (de Lau and Breteler, 2006). It is characterized by motor symptoms such as bradykinesia, rigidity, tremors, and postural instability, as well as non-motor symptoms including mood disorders, autonomic dysfunction, and cognitive impairment (Pagano et al., 2016). Cognitive impairment is observed in approximately one-third of newly diagnosed PD patients and becomes more prevalent over time (Broeders et al., 2013). Around 50% of PD patients show signs of mild cognitive impairment (MCI) by the fifth year after diagnosis, with approximately 26% progressing to PD-related dementia (PDD) within the subsequent 5 years (de Lau and Breteler, 2006).

Patients with MCI and PDD experience poor quality of life (Lawson et al., 2016). Therefore, it is imperative to identify accessible biomarkers that can reflect the severity of cognitive impairment in PD. One potential biomarker, neurofilament light chain (NfL), has recently emerged as a key indicator of neurodegeneration (Khalil et al., 2018). Neurofilaments are abundant proteins expressed in neurons and belong to the intermediate filament family. The subunits of neurofilaments include NfL, neurofilament medium chain, neurofilament heavy chain, alpha-internexin, and peripherin (Khalil et al., 2018). Among these subunits, NfL is the most commonly used biomarker. The release of neurofilaments into the cerebrospinal fluid (CSF) is considered a specific indicator of neuronal damage in neurodegenerative conditions (Gaiottino et al., 2013). Previous studies have demonstrated a strong correlation between CSF and blood NfL levels, making blood NfL a favorable alternative to CSF biomarkers (Rohrer et al., 2016). Blood NfL levels have shown potential for distinguishing PD from atypical parkinsonian syndromes (Bridel et al., 2019). Longitudinal studies have also associated increased NfL levels in blood before or around disease onset with the risk of developing PD and various aspects of disease progression, including declining performance on motor assessment scales and cognitive tests (Mollenhauer et al., 2020; Wilke et al., 2020).

In this study, our hypothesis is that the levels of blood-derived NfL and its accumulation during the PD progression could serve as a potential biomarker for predicting subsequent cognitive decline. To investigate this, we conducted a study utilizing a large single-center cohort. We set out to determine whether the levels of serum NfL (1) exhibit differences between patients with cognitively normal PD (PD-CN) and those without PD (normal controls, NC), (2) increase as PD advances, and (3) have the ability to predict long-term cognitive deterioration in individuals with PD.

## 2 Materials and methods

### 2.1 Participants and clinical evaluation

The study employed a prospective, longitudinal design and recruited participants from Tianjin Huanhu Hospital between September 2017 and September 2019. PD patients were included based on the following criteria: (1) aged between 40 and 85 years old, of Chinese Han ethnicity; (2) PD diagnosis independently confirmed by two neurologists using the 2015 Movement Disorder Society (MDS) Clinical Diagnosis Criteria for PD (Postuma et al., 2015). Exclusion criteria consisted of: (1) a history of other neuropsychiatric diseases; (2) an uncertain PD diagnosis; (3) major medical conditions. PD-CN was defined as a clinical diagnosis of PD with no cognitive complaints and normal cognitive performance, indicated by a Montreal Cognitive Assessment (MoCA) score of ≥26 points. Age, gender, body mass index (BMI), and education level-matched NC were included from the physical examination center of Tianjin Huanhu Hospital. The Human Participants Ethics Committee of Tianjin Huanhu Hospital approved the study (No. 2016-026), and all participants provided written informed consent. Initially, the study included 193 participants, consisting of 106 PD-CN patients and 87 NC individuals. The study adhered to the Declaration of Helsinki. Global cognitive function was assessed using the MoCA test, while memory was evaluated using the Hopkins Verbal Learning Test-Delayed Recall (HVLT-DR). Information processing speed was assessed using the Digit Symbol Substitution Test (DSST) and Trail Making Test (TMT) A. Language function was assessed using the Semantic Fluency Test (SFT), and visuospatial function was evaluated using the Clock Drawing Test (CDT). Executive function was assessed using TMT B.

### 2.2 Definitions of cognitive impairment

In this study, a MoCA cutoff score of <26 was used to diagnose PD-CI (Dalrymple-Alford et al., 2010; Kasten et al., 2010). PDD was defined based on the Clinical diagnostic criteria for dementia associated with Parkinson’s disease (Emre et al., 2007). A clinical diagnosis of PDD was made when deficits in at least two cognitive domains were severe enough to impact daily life and normal functioning, with a MoCA cutoff score of <21 (Dalrymple-Alford et al., 2010; Kasten et al., 2010). PD patients were followed up for clinical assessment at an average of 2 ± 0.6 years. At the follow-up time, serum NfL levels were measured in 93 PD patients (65 PD-CN patients, 25 PD-MCI patients, 3 PDD patients) and 68 NC individuals (56 NC-CN, 12 NC-MCI patients). PD-MCI and PDD patients were grouped together as PD-CI due to the low number of PDD patients in this study.

### 2.3 Measurement of NfL

At enrollment, 10 mL of peripheral blood was collected from each participant prior to clinical evaluation. Within 1 h of collection, the blood samples were centrifuged at 2,500 g for 15 min and then stored at −80°C for less than 3 months before testing. Serum NfL concentrations were measured by investigators who were blinded to the clinical diagnosis. The serum samples were transferred onto the single molecule array (Simoa) platform using a NfL assay kit (Quanterix; Lexington, MA), as previously described (Hansson et al., 2017). To reduce the risk of any potential bias, the analysts conducting the assays were blinded to patient status from each sample.

### 2.4 Statistical analysis

Statistical analysis was performed using SPSS 21.0 software. Continuous variables were presented as mean value ± standard deviation (SD). At baseline, independent samples *t*-tests were conducted for normally distributed data (age, BMI, education level, and baseline NfL) to compare PD-CN patients and NC individuals. Repeated-measures analysis of variance (RMANOVA) with *post hoc* simple main effect analysis was used to compare serum NfL levels and MoCA scores between PD-CN patients and NC individuals at baseline and follow-up time. The evaluation time point (follow-up time versus baseline) was considered the within-subject factor, while the groups (PD-CN versus NC) were considered the between-subject factor. Logistic regression analyses were performed to evaluate the correlation between serum NfL and categorical variables (PD-CI versus PD-CN). According to previous studies (Baiano et al., 2020; Miller and Cronin-Golomb, 2010; Pilotto et al., 2016; Weintraub et al., 2022), cognitive impairment in PD was associated with age, gender, history of hypertension, history of diabetes, history of atrial fibrillation, history of prior myocardial infarct, history of prior stroke, BMI, education level, disease duration, baseline Unified Parkinson’s Disease Rating Scale (UPDRS) III scores, baseline Hoehn and Yahr (H&Y), baseline MoCA scores, and levodopa-equivalent daily dose (LEDD). Hazard ratio (HR) and corresponding 95% confidence intervals (CI) were calculated for the model adjusted for age, gender, history of hypertension, history of diabetes, history of atrial fibrillation, history of prior myocardial infarct, history of prior stroke, BMI, education level, disease duration, baseline Unified Parkinson’s Disease Rating Scale (UPDRS) III scores, baseline Hoehn and Yahr (H&Y), baseline MoCA scores, and levodopa-equivalent daily dose (LEDD). Receiver operating characteristic (ROC) curve analysis and Youden’s index were used to determine the optimal cut-point for serum NfL in PD diagnosis and predicting clinical conversion to MCI or dementia in PD-CN.

## 3 Results

### 3.1 Baseline demographic data, motor and cognitive function

**FIGURE 1:**
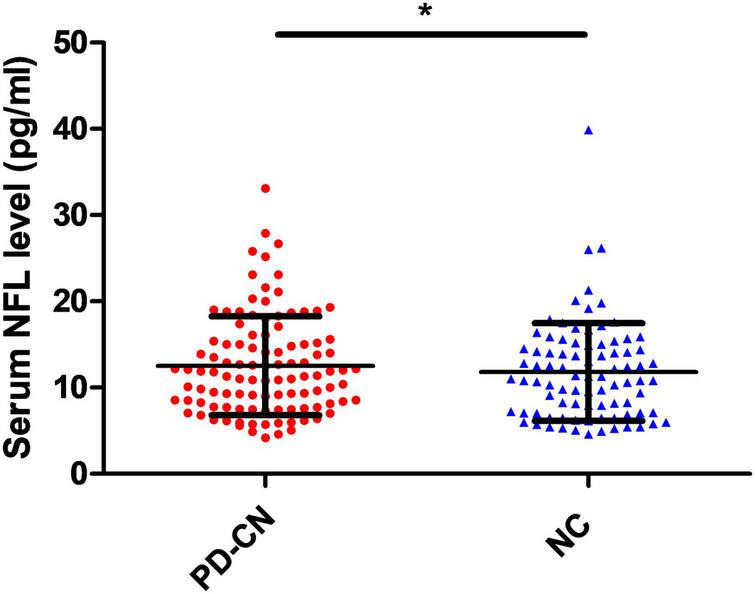
Mean ± SD concentrations of serum NfL levels in PD-CN patients and NC. NC, normal controls; NfL, neurofilament light chain; PD-CN, cognitively normal Parkinson’s disease; SD, standard deviation. *p < 0.05.

Table 1 presented the demographic data, motor, and cognitive function of the participants. The results indicate that there were no significant differences in age, gender, history of hypertension, history of diabetes, history of atrial fibrillation, history of prior myocardial infarct, history of prior stroke, BMI, education level, or MoCA scores between PD-CN patients and NC (all *p* > 0.05, Table 1). However, the serum NfL levels were significantly higher in PD-CN patients (13.01 ± 5.84 pg/mL) compared to NC (11.23 ± 5.40 pg/mL; Figure 1). The area under the curve (AUC) for distinguishing PD-CN from NC was found to be 0.538 (95% CI 0.456–0.620) (Supplementary Figure 1).

**TABLE 1:** Baseline demographic data and clinical assessments for all participants.

Indicators	PD-CN (*n* = 106)	NC (*n* = 87)	*p*-value
Age (years), mean ± SD	62.32 (8.77)	61.96 (8.67)	0.779^a^
Gender, male/female	74/32	53/34	0.195^b^
Hypertension, yes/no	32/74	29/58	0.640^b^
Diabetes, yes/no	17/89	17/70	0.525^b^
Atrial fibrillation, yes/no	14/92	9/78	0.541^b^
Prior myocardial infarct, yes/no	12/94	7/80	0.447^b^
Prior stroke, yes/no	11/95	6/81	0.396^b^
BMI (kg/m^2^), mean ± SD	23.54 (3.01)	23.69 (2.57)	0.710^a^
Educational level (years), mean ± SD	10.81 (2.28)	10.90 (2.35)	0.799^a^
Disease duration (years), mean ± SD	4.87 (1.95)		
UPDRS III (points), mean ± SD	20.51 (8.63)		
H&Y (stage), mean ± SD	2.25 (0.753)		
MoCA (points), mean ± SD	27.93 (1.22)	28.26 (1.21)	0.062^a^
HVLT-DR (points), mean ± SD	46.84 (9.31)	48.43 (8.64)	0.534^a^
DSST (points), mean ± SD	41.64 (9.30)	45.74 (8.72)	0.234^a^
SFT (points), mean ± SD	13.32 (3.85)	13.56 (3.78)	0.435^a^
CDT (points), mean ± SD	9.5 (1.1)	9.6 (0.6)	0.342^a^
TMT A (seconds), mean ± SD	31.5 (15.3)	30.6 (12.5)	0.213^a^
TMT B (seconds), mean ± SD	70.5 (36.8)	67.8 (35.7)	0.345^a^
LEDD (mg/d), mean ± SD	212.23 (323.62)		
Serum NfL level (pg/mL)	13.01 (5.84)	11.23 (5.40) pg/ml	0.031^a^ *

BMI, body mass index; H&Y, Hoehn and Yahr; LEDD, levodopa-equivalent daily dose; MoCA, Montreal Cognitive Assessment; NC, normal controls; NfL, neurofilament light chain; PD, Parkinson’s disease; PD-CN, cognitively normal PD; SD, standard deviation; UPDRS, Unified Parkinson’s disease.

*^a^*independent samples *t*-test;

*^b^*Chi-square test;

*p-value < 0.05.

### 3.2 Comparison in serum NfL level, MoCA between PD-CN patients and NC at baseline and follow-up time

**FIGURE 2:**
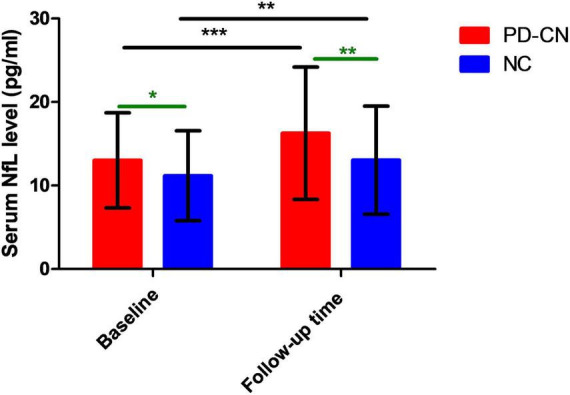
Serum NfL levels in PD-CN patients and NC at baseline and follow-up time. NC, normal controls; NfL, neurofilament light chain; PD-CN, cognitively normal Parkinson’s disease. *p < 0.05, **p < 0.01, ***p < 0.001.

Significant main effects of evaluation time point (follow-up time versus baseline) and group (PD-CN versus NC) were observed on the serum NfL levels [evaluation time point: *F*(1, 159) = 35.934, *p* < 0.001; group: *F*(1, 159) = 7.133, *p* = 0.008]. After conducting Bonferroni post-hoc analysis, it was found that both PD-CN patients and NC at follow-up time (PD-CN patients: 16.26 ± 7.92 pg/mL; NC: 13.04 ± 6.48 pg/mL) exhibited higher serum NfL levels compared to those at baseline (Figure 2). Additionally, the serum NfL levels were significantly higher in PD-CN patients than in NC at both baseline and follow-up time (Figure 2).

**FIGURE 3:**
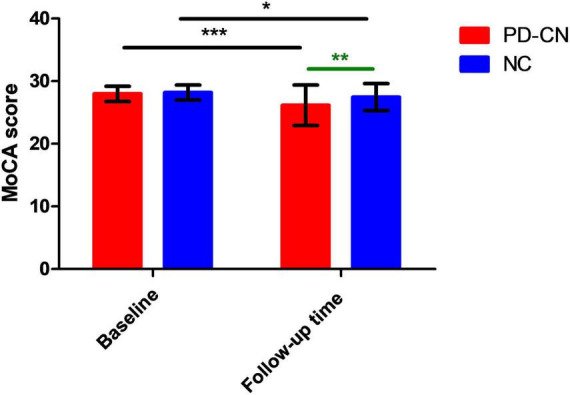
MoCA scores in PD-CN patients and NC at baseline and follow-up time. MoCA, Montreal Cognitive Assessment; NC, normal controls; PD-CN, cognitively normal Parkinson’s disease. *p < 0.05, **p < 0.01, ***p < 0.001.

Significant main effects of evaluation time point (follow-up time versus baseline) and group (PD-CN versus NC) were also observed on MoCA scores [evaluation time point: *F*(1, 159) = 29.107, *p* < 0.001; group: *F*(1, 159) = 8.820, *p* = 0.003]. After conducting Bonferroni post-hoc analysis, it was found that both PD-CN patients and NC at follow-up time (PD-CN patients: 26.15 ± 3.24 points; NC: 27.44 ± 2.17 points) exhibited lower MoCA scores compared to those at baseline (Figure 3). Additionally, the MoCA scores were significantly lower in PD-CN patients than in NC at both baseline and follow-up time (Figure 3).

### 3.3 Prediction of conversion to MCI or dementia based on serum NfL levels in PD

Among the 93 PD-CN patients at the follow-up time, 28 PD patients exhibited cognitive impairment, while 12 patients showed cognitive impairment among the 68 NC participants at the follow-up time. There was no significant difference in the rate of cognitive impairment conversion between PD patients and NC (χ^2^ = 3.266, *p* = 0.071). However, PD patients with high serum NfL levels were found to have a higher likelihood of transitioning from normal cognition to MCI or dementia (HR 1.107, 95% CI 1.010–1.213, *p* = 0.030; Supplementary Table 1) in logistic regression analysis, after adjusting for various factors including age, gender, history of hypertension, history of diabetes, history of atrial fibrillation, history of prior myocardial infarct, history of prior stroke, BMI, education level, disease duration, baseline UPDRS III scores, baseline H&Y, baseline MoCA scores, and LEDD.

**FIGURE 4:**
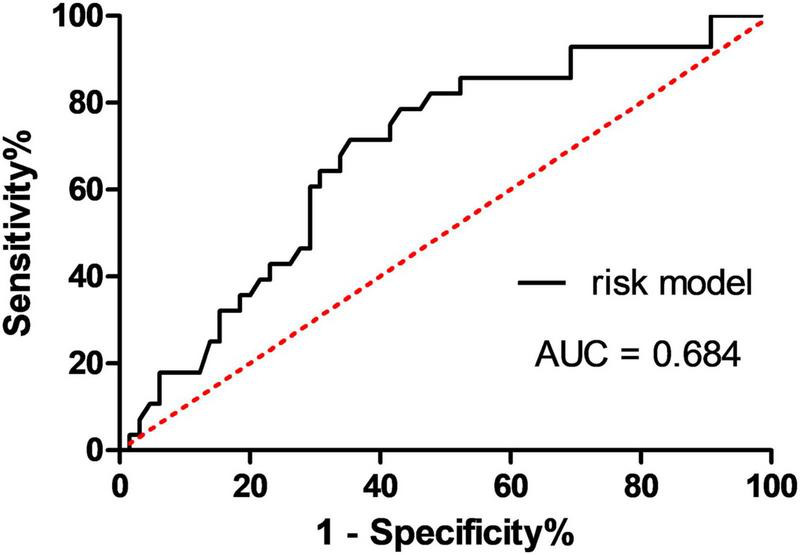
Receiver operating characteristic curves for predicting PD-CN conversion to MCI or dementia at follow-up time using the serum NfL level. AUC, area under the curve; MCI, mild cognitive impairment; PD-CN, cognitively normal Parkinson’s disease.

The AUC for PD-CN conversion to MCI or dementia at follow-up time was determined to be 0.684 (95% CI 0.569–0.799), and the optimal cut-point for serum NfL levels was identified as 12.65 pg/mL (with a sensitivity of 71.4% and specificity of 64.6%), as illustrated in Figure 4.

## 4 Discussion

The present study yielded several important findings. Firstly, it was observed that serum NfL levels were higher in PD-CN patients compared to NC individuals. This finding is in line with recent studies (Chen et al., 2020; Hansson et al., 2017) that have reported similar results. However, it is worth noting that some studies have indicated no significant difference in blood NfL levels between PD patients and NC individuals (Lin et al., 2018; Marques et al., 2019). A recent study reported that there was no statistically significant difference in plasma NfL concentrations between PD and NC groups (Batzu et al., 2022). Zarkali et al. (2024) found that plasma NFL did not statistically differ between people with PD and control participants. A meta-analysis also revealed no differences in blood NfL levels in PD patients when not stratified by disease severity compared to NC individuals (Wang et al., 2019). More recent studies, which have stratified PD patients based on disease duration and stage, have shown that advanced PD patients tend to have higher blood NfL levels compared to those in early disease stages (Lin et al., 2019; Niemann et al., 2021). For instance, Lin et al. (2019) suggested a plasma NfL cutoff value of 12.34 pg/mL for distinguishing between PD patients and NC individuals in advanced stages, with a modest sensitivity of 53.2% and high specificity of 90.5%. The present study found that serum NfL levels did not have diagnostic value for PD-CN (AUC 0.538, 95% CI 0.456–0.620). This modest result may be attributed to the analysis not being stratified by disease severity. Therefore, it is crucial to conduct more large-scale studies that stratify participants based on disease severity in order to thoroughly investigate the diagnostic value of serum NfL levels for differentiating PD patients from NC individuals.

The present study observed an association between PD progression and increased serum NfL levels. This finding aligns with recent studies that have also reported significant longitudinal increases in serum NfL levels among PD patients (Urso et al., 2023; Yang et al., 2023). These consistent findings suggest that serum NfL levels may serve as a potential biomarker for PD. However, it is important to note that elevated NfL levels have also been observed in other neurodegenerative diseases (Mattsson et al., 2017). A recent meta-analysis further demonstrated increased NfL concentrations in serum and plasma among patients with Alzheimer’s disease (AD) and frontotemporal dementia (FTD), when compared to individuals without cognitive impairments (Gu et al., 2023). In addition to investigating the diagnostic value of serum NfL levels in PD, this study aimed to explore the clinical significance of serum NfL levels in predicting longitudinal cognitive impairment in individuals with PD.

The study findings revealed that serum NfL levels can serve as a predictive marker for cognitive impairment in individuals with PD over a 2-year period. The AUC for the conversion of PD-CN to MCI or dementia was determined to be 0.684 (95% CI 0.569–0.799). These results are consistent with several recent studies. For instance, Ma et al. (2021) and Niemann et al. (2021) demonstrated that higher baseline NfL levels were predictive of cognitive outcomes in PD. Similarly, Buhmann et al. (2022) confirmed that age-adjusted serum NfL levels were indicative of cognitive decline in PD. Chen et al. (2020) found that elevated plasma NfL levels were predictive of incident dementia in PD. Additionally, Lin et al. (2019) reported that higher baseline NfL levels were associated with a faster rate of cognitive decline in PD. Aamodt et al. (2021) discovered that PD participants with elevated plasma NfL levels were more likely to develop incident cognitive impairment. In addition, Batzu et al. (2022) reported that baseline plasma NfL predicted Mini-Mental State Examination (MMSE) decline over time in the PD group. Zarkali et al. (2024) found that mean plasma NfL was correlated with cognition (combined cognitive score) both at baseline (*r* = −0.246, *p* = 0.037) and after 3-year follow-up (*r* = −0.223, *p* = 0.040). Collectively, these studies provide further support for the significance of blood NfL levels in predicting cognitive decline. A serum NfL measurement may help neurologists in identifying PD at risk of cognitive impairment progression and may have the potential for early treatment of these individuals.

The specific pathophysiological mechanisms that link NfL to cognitive dysfunction in PD remain unclear. Cognitive decline in PD is caused by multiple pathological mechanisms that ultimately result in cortical-subcortical dysfunction (Aarsland et al., 2017). Previous studies have found a correlation between macro- and microstructural changes and cognitive decline in PD patients. One study observed a relationship between increased levels of NfL and damage to gray and white matter in PD (Rektor et al., 2018). In patients with PDD, the degeneration of dopaminergic neurons in the midbrain substantia nigra may be accompanied by axonal degeneration (Papuć and Rejdak, 2020). A neuroimaging study has suggested that the observed increase in NfL levels in the cerebrospinal fluid (CSF) of PDD patients may be due to axonal injury or loss (Rektor et al., 2018). NfL levels in the CSF of PD patients are correlated with cognitive indicators such as Aβ1-42, tau, phosphotau, and α-synuclein (Sampedro et al., 2020). Increased levels of plasma α-synuclein have also been associated with reduced cortical thickness in certain brain regions (Chen et al., 2020). Additionally, in a mouse model of PD, higher levels of NfL in the CSF and plasma are positively correlated with the number and size of neuronal α-synuclein inclusions (Bacioglu et al., 2016).

However, our study has some limitations. Firstly, the PD patients in our cohort had a median disease duration of 4.87 years at the time of biofluid sampling, so our findings may not be applicable to earlier stages of the disease. Secondly, our analysis using ROC demonstrated only modest predictive performance for serum NfL alone in determining the conversion from normal cognition to MCI or dementia on an individual basis. This suggests that incorporating serum NfL into a multi-marker panel may be necessary for more accurate prediction of clinical conversion. Thirdly, the diagnosis of PD-MCI in this study did not rely on assessments across various cognitive domains. Instead, a multifaceted approach was employed, utilizing the MoCA to gauge overall cognitive performance. Specific cognitive domains were also targeted with the following tests: the DSST and TMT A to assess information processing speed; the SFT to evaluate language abilities; the CDT to assess visuospatial skills; and TMT B to assess executive functions. This methodological choice was deliberate and made prior to data collection for several reasons: Relying on a single test per domain could potentially diminish the sensitivity to detect cognitive impairments; a reduced sensitivity increases the likelihood of a type II error, which occurs when a true effect is incorrectly concluded to be absent. Fourthly, the study includes a large single-center cohort. The single-center study showed the potential limitations of geographical or demographic biases. This limited the potential for external promotion of our findings in a multicenter study or with a different patient population. Lastly, the study follows patients for an average of 2 ± 0.6 years. It is unclear whether this timeframe is sufficient to draw conclusions about the predictive value of NfL for cognitive decline. Longer-term follow-up might provide more robust data.

## 5 Conclusion

In conclusion, our study found that PD patients have significantly higher levels of serum NfL compared to individuals without PD. Furthermore, serum NfL levels increase as PD progresses and can predict cognitive impairment within a 2-year timeframe. Serum NfL may serve as a feasible, non-invasive biomarker of cognitive progression in PD. However, further studies and functional experiments are needed to validate these findings.

## Data Availability

The original contributions presented in this study are included in this article/Supplementary material, further inquiries can be directed to the corresponding authors.
