# Supplementary material for: Predictive value of serum neurofilament light chain for cognitive impairment in Parkinson’s disease
Source: Front Aging Neurosci. 2024 Dec 5;16:1465016. doi: 10.3389/fnagi.2024.1465016 (PMC11655485; doi:10.3389/fnagi.2024.1465016)
Supplement: Supplementary file 1 [file Data_Sheet_1.docx]

Supplementary Table 1. Association between baseline demographic data, motor, cognitive function, serum NfL levels and risk of conversion to MCI or dementia in PD.

| Indicators | Conversion to MCI or dementia in PD | |
| --- | --- | --- |
|  | HR (95% CI) | *p* value |
| Serum NfL level | 1.107 (1.010-1.213) | 0.030^*^ |
| Age | 1.067 (1.002, 1.137) | 0.043^*^ |
| Gender | 1.242 (0.351, 4.401) | 0.737 |
| Hypertension | 2.671 (0.501, 14.237) | 0.250 |
| Diabetes | 0.379 (0.017, 8.235) | 0.537 |
| Atrial fibrillation |  | 0.999 |
| Prior myocardial infarct |  | 0.999 |
| Prior stroke |  | 0.999 |
| BMI | 0.935 (0.779, 1.124) | 0.476 |
| Educational level | 1.011 (0.802, 1.273) | 0.929 |
| Disease duration | 0.945 (0.716, 1.246) | 0.687 |
| Baseline UPDRS III | 1.072 (1.001, 1.148) | 0.048 |
| Baseline H&Y | 1.131 (0.563, 2.272) | 0.730 |
| Baseline MoCA | 0.755 (0.491, 1.161) | 0.200 |
| LEDD | 1.001 (0.702, 1.352) | 0.975 |

Abbreviations: BMI, body mass index; CI, confidence interval; HR, hazard ratio; H&Y, Hoehn and Yahr; LEDD, levodopa-equivalent daily dose; MCI, mild cognitive impairment; MoCA, Montreal Cognitive Assessment; NfL, neurofilament light chain; PD, Parkinson's disease; SD, standard deviation; UPDRS, Unified Parkinson's disease. ^*^, *p* < 0.05.


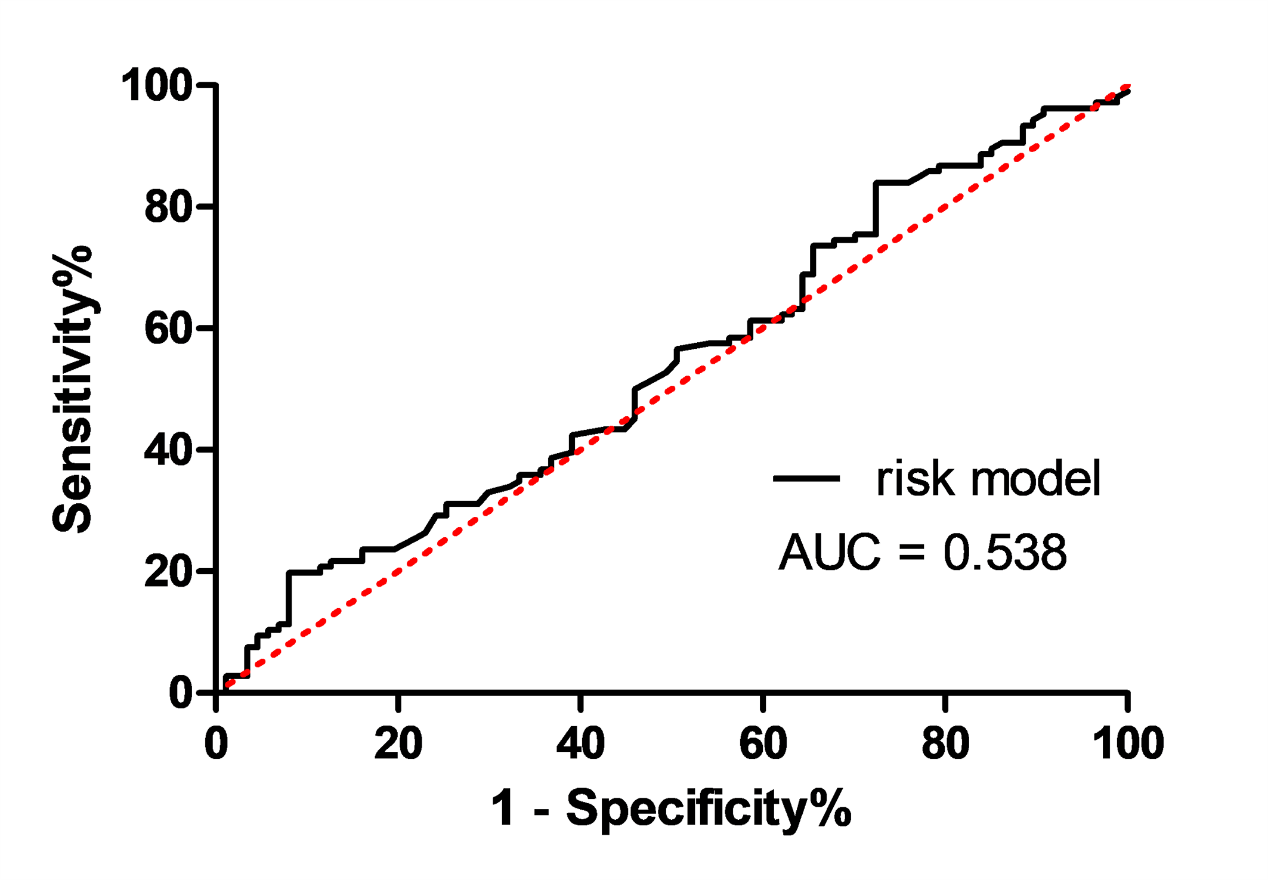


Supplementary Figure 1. Receiver operating characteristic curves for distinguishing PD-CN from NC using the serum NfL level. Abbreviations: AUC, area under the curve; MCI, mild cognitive impairment; PD-CN, cognitively normal Parkinson's disease.
